# The Moderating Role of Interoceptive Awareness in the Relationship Between Difficulties in Emotion Regulation and Somatic Symptoms

**DOI:** 10.3390/bs16101884

**Published:** 2026-10-13

**Authors:** Erinç Erbildim

**Affiliations:** Faculty of Humanities and Social Sciences, Cyprus Aydın University, Kyrenia 99320, Cyprus; erincerbildim@cau.edu.tr

**Keywords:** emotion-regulation difficulties, somatic symptoms, interoceptive awareness

## Abstract

Difficulties in emotion regulation have been associated with greater somatic symptom severity. However, the role of interoceptive awareness in this relationship remains unclear. This study examined whether overall interoceptive awareness and its specific dimensions moderate the association between difficulties in emotion regulation and somatic symptoms. The sample comprised 394 adults recruited from the general population through online participant recruitment platforms. Participants completed the Difficulties in Emotion Regulation Scale-16, Somatic Symptom Scale-8, and Multidimensional Assessment of Interoceptive Awareness. Moderation analyses were conducted using PROCESS Model 1, controlling for age, gender, and chronic physical illness. These findings showed that difficulties in emotion regulation were positively associated with somatic symptoms (r = 0.53, *p* < 0.001). Overall interoceptive awareness significantly moderated this association (B = 0.069, SE = 0.020, *p* < 0.001, ΔR^2^ = 0.020), such that the association became stronger at higher levels of interoceptive awareness. Following Bonferroni correction, significant moderating effects were also observed for noticing, emotional awareness, self-regulation, and body listening, whereas not distracting, not worrying, attention regulation, and trusting were not significant. Overall, the findings suggest that greater interoceptive awareness may be associated with a stronger relationship between emotion regulation difficulties and somatic symptoms.

## 1. Introduction

In emotion regulation affective experiences are modulated by cognitive, behavioural, interpersonal, and intrapersonal strategies (Gross, 2002). According to the comprehensive conceptualisation of Gratz and Roemer (2004), emotion regulation includes several facets: (a) emotional awareness; (b) acceptance of emotions; (c) the ability to proceed with goal-directed behaviour when experiencing negative emotions; and (d) the ability to flexibly apply emotion regulation strategies to meet individual goals and situational demands.

Cole et al. (2019) defined difficulties in emotion regulation as the “inability to modulate emotional responses according to situational demands”. They noted that they involve maladaptive strategies such as worry and rumination, which hinder emotion regulation (Mansueto et al., 2022). Emotion-regulation difficulties are associated with several psychopathologies and mental health conditions, including depression (Wu et al., 2024), anxiety (Korkmaz et al., 2025), social anxiety disorder (Werkmann & Schwenck, 2026), and somatic symptoms (Erbildim & Nweke, 2025).

Fonagy and Allison (2012) explain that a lack of emotion regulation skills may lead to the repression and avoidance of emotional experiences. These maladaptive approaches are associated with somatising affective experiences rather than verbal communication and expression.

Somatic symptoms are physical symptoms that may or may not have an organic basis (Fink & Rosendal, 2008). The absence of an organic basis does not imply that these symptoms are not real or do not exist. For example, headaches and dizziness are common, especially during stressful periods. However, these symptoms are not always attributable to medical conditions such as a brain tumour or migraine and are considered temporary. Perez and Szabo (2025) note that somatic symptoms are linked to psychological distress and trauma; individuals seeking mental health care often report physical symptoms. Somatic symptoms may reduce overall quality of life (Huang et al., 2025), impair mental and physical well-being (Barends et al., 2025), and affect the outcome of psychological treatment (Huijbregts et al., 2013).

The perseverative cognition hypothesis suggests that bodily symptoms are more common during stressful periods. According to the hypothesis, the duration and intensity of associations are attributed to a lack of emotional regulation strategies for coping with psychological distress, rather than a direct effect of stress on somatic symptoms (Brosschot et al., 2006). Selvi and Bozo (2023) also indicate that difficulties in emotion regulation moderate the relationship between perceived stress and somatic symptoms. In the same study, somatic symptom severity was positively correlated with total emotion regulation difficulty scores and their subscales. Similarly, Schnabel et al. (2022) reported that patients with somatic symptom disorder obtained higher scores on difficulties in emotion regulation and were less likely to use adaptive emotion regulation strategies. According to Lee et al. (2024), attention regulation and cognitive reappraisal, which are adaptive emotion regulation strategies, are negatively associated with somatic symptom severity.

Difficulties in emotion regulation have consistently been identified as a factor associated with somatic symptoms. Individuals who lack adaptive emotion regulation strategies may be less able to process emotional experiences effectively, increasing the likelihood that somatic symptoms are expressed. However, individuals with similar levels of emotion dysregulation do not necessarily report the same degree of somatic complaints. This variability suggests that additional mechanisms may influence the relationship between emotion-regulation difficulties and somatic symptoms. One promising candidate is interoceptive awareness. While interoception is defined as “the process by which the nervous system senses, interprets, and integrates signals originating from within the body, providing a moment-by-moment mapping of the body’s internal landscape across conscious and unconscious levels” (Khalsa et al., 2018, p. 501), interoceptive awareness refers to the conscious level of interoception with multiple dimensions available to self-report (Mehling et al., 2018).

The multidimensional conceptual framework for interoceptive awareness proposed by Mehling et al. (2012) comprises five broad theoretical dimensions: awareness of body sensations; emotional reaction and attentional response to sensations; capacity to regulate attention; awareness of mind–body integration; and trusting body sensations. These theoretical dimensions are operationalized through eight MAIA subscales. Specifically, awareness of body sensations is represented by Noticing (awareness of bodily sensations); emotional reaction and attentional response to sensations by Not-Distracting (tendency not to ignore or distract from discomfort) and Not-Worrying (tendency not to experience emotional distress in response to discomfort); capacity to regulate attention by Attention Regulation (ability to sustain and control attention to bodily sensations); awareness of mind–body integration by Emotional Awareness (awareness of the connection between bodily sensations and emotions), Self-Regulation (ability to regulate distress through attention to bodily sensations), and Body Listening (active listening to the body for insight); and trusting body sensations by Trusting (experiencing the body as safe and trustworthy). Thus, the five dimensions represent the broader conceptual domains of interoceptive awareness, whereas the eight MAIA subscales represent the specific measurable facets of these domains (Mehling et al., 2012).

Interoceptive awareness is linked to better emotion regulation capacity, as emotion processing requires identifying and being aware of emotions (Kever et al., 2015). Damasio (1994) proposes the somatic marker hypothesis, which holds that bodily signals are essential for understanding feelings and making decisions. The hypothesis emphasises that recognising the physiological changes associated with emotions is crucial for regulating them through physiological responses. Lazzarelli et al. (2024) found that improving interoceptive ability through mind–body interventions such as mindfulness may contribute to greater emotion regulation capacity. Türkel et al. (2025) reported a negative relationship between interoceptive awareness and emotion regulation difficulties, suggesting that greater interoceptive awareness is associated with fewer emotion regulation difficulties.

The literature on the relationship between interoceptive awareness and somatic symptoms is mixed. Some studies report that greater interoceptive awareness is linked to fewer physical symptoms, whereas others report a positive relationship between the two variables. For instance, Price et al. (2025) observed that improving interoceptive sensibility through Mindful Awareness in Body-Oriented Therapy (MABT) was associated with reduced pain interference. Similarly, Arey et al. (2024) found that improved interoceptive awareness through a brief mindfulness self-compassion intervention was associated with reduced pain among patients with fibromyalgia. Lee et al. (2024) examined the independent contributions of emotion-regulation strategies and dimensions of interoceptive awareness to depression, anxiety, and somatization among clinical outpatients with depression and anxiety disorders. The study indicated that among the MAIA subscales, attention regulation was negatively related to somatization, while other dimensions had no significant relationship. The present study extends this work by examining overall interoceptive awareness and its specific dimensions as a moderator of the association between emotion-regulation difficulties and somatic symptoms in a non-clinical adult sample. Rather than examining the additive predictive roles of emotion-regulation strategies and interoceptive awareness, the current study examines the interaction between emotion-regulation difficulties and interoceptive awareness in predicting somatic symptoms.

In contrast, other studies have reported that higher interoceptive awareness is linked to perceiving physical symptoms more severely. Bogaerts et al. (2022) found that patients with fibromyalgia and chronic fatigue syndrome scored higher on sensitivity to bodily sensations and attention to negative sensations. Likewise, Todd et al. (2024) concluded that self-reported interoceptive awareness was positively correlated with fibromyalgia symptom severity.

These contradictory findings may partly be explained by the context-dependent nature of interoceptive awareness. Hanley et al. (2017) noted that interoceptive awareness characterised by anxiety-related hypervigilance may be maladaptive. Similarly, Trevisan et al. (2021) suggest that although attention to bodily signals may facilitate the recognition and regulation of internal states, excessive attention and worry toward bodily sensations may increase anxiety and the salience of bodily sensations. Thus, interoceptive awareness may not be uniformly adaptive.

The association between emotion-regulation difficulties and somatic symptoms has been consistently examined. However, limited research has examined whether interoceptive awareness moderates the association between emotion-regulation difficulties and somatic symptoms. Interoceptive awareness influences the perception and interpretation of internal bodily signals accompanying emotional arousal; thus, it may alter the extent to which emotion-regulation difficulties are translated into somatic symptom experiences. Adaptive interoceptive awareness may act as a buffering mechanism; it contributes to emotion regulation by facilitating emotional and bodily awareness; thus, fewer somatic symptoms are expected in relation to emotion-regulation difficulties. Conversely, maladaptive interoceptive awareness, characterised by hypervigilance or excessive attention to bodily sensations, may increase the salience of bodily signals and amplify this association. Thus, interoceptive awareness may plausibly attenuate or intensify the relationship between emotion-regulation difficulties and somatic symptoms.

The present study aimed to examine the relationship between difficulties in emotion regulation and somatic symptoms and to assess the moderating role of interoceptive awareness. Given the multidimensional nature of interoceptive awareness, the moderating roles of the individual MAIA dimensions were also examined. As the existing literature does not provide sufficient evidence to specify directional predictions for individual MAIA dimensions, these subscale analyses were considered exploratory. The hypotheses of the study are as follows:

**H1.**  *Difficulties in emotion regulation are positively associated with somatic symptoms.*

**H2.**  *Total interoceptive awareness and its eight dimensions (noticing, not distracting, not worrying, attention regulation, emotional awareness, self-regulation, body listening, and trusting) moderate the relationship between difficulties in emotion regulation and somatic symptoms.*

## 2. Materials and Methods

### 2.1. Participants

A total of 397 survey submissions were received from participants in the United States recruited via the participant recruitment platforms Prolific and Positly using a convenience sampling method between 17 July and 27 July 2026. The inclusion criterion was being aged 18 years or older. Responses with completion times below the predetermined minimum criterion were excluded. Accordingly, three submissions were excluded, resulting in a final analytic sample of 394 participants. To further ensure data quality, duplicate participation from the same IP address was prevented, and all survey items required a response before submission; therefore, the final dataset had no missing responses. Participants who completed the survey were compensated through the platform.

For sample size calculation, an a priori power analysis was conducted using GPower 3.1 for hierarchical multiple regression. Assuming a medium effect size (f^2^ = 0.15 (Cohen, 1988), an alpha level of 0.05, and a statistical power of 0.80, the final sample size exceeded the minimum required. Participation was voluntary, and participants could withdraw from the study at any time. The informed consent form was presented before participants answered the survey questions.

The sample had a mean age of 34.85 years (SD = 12.31; range = 19–78 years). Of the participants, 39.3% were female (n = 155) and 60.7% were male (n = 239). Regarding educational attainment, 20.6% had completed high school, 54.3% held a bachelor’s degree, 22.6% had a master’s degree, and 2.5% held a doctoral degree. Regarding perceived income, 26.9% of participants reported a low income, 67.3% reported an average income, and 5.8% reported a high income. Marital status was distributed as follows: 58.6% were single, 36.0% were married, 5.1% were divorced, and 0.3% were widowed. Finally, 20.1% of participants reported having a chronic physical illness, whereas 79.9% reported no history of chronic physical illness.

### 2.2. Research Tools

#### 2.2.1. Sociodemographic Information Form

The sociodemographic information form was developed by the researcher and used to collect data on participants’ demographic characteristics. The form included questions regarding age, gender, educational attainment, marital status, income level, and the presence of chronic illness. These variables were used to describe the sample, and selected demographic characteristics were included as covariates in the analyses.

#### 2.2.2. Difficulties in Emotion Regulation Scale-16 (DERS-16)

The Difficulties in Emotion Regulation Scale-16 (DERS-16) was used to assess difficulties in emotion regulation. The scale has 16 items rated on a 5-point Likert scale with responses ranging from 1 (almost never) to 5 (almost always). A total score was calculated by summing the 16 item scores, with higher scores indicating greater difficulties in emotion regulation. The DERS-16 has excellent internal consistency, with Cronbach’s alpha coefficients ranging from 0.92 to 0.95. The scale also demonstrated good convergent and discriminant validity across one clinical and two community samples, based on its associations with related constructs (Bjureberg et al., 2016). In the present study, the scale demonstrated excellent internal consistency (Cronbach’s α = 0.95; McDonald’s ω = 0.95).

#### 2.2.3. Somatic Symptom Scale-8 (SSS-8)

Somatic symptom severity was assessed using the Somatic Symptom Scale-8 (SSS-8). The SSS-8 consists of eight items, which report somatic symptoms experienced during the past seven days. Each item is rated on a 5-point Likert scale ranging from 0 (not at all) to 4 (very much). A total score was calculated by summing the eight item scores, yielding a possible range of 0–32, with higher scores indicating greater somatic symptom severity. The SSS-8 has demonstrated good internal consistency, with a Cronbach’s alpha coefficient of 0.81 and good construct validity, with greater somatic symptom burden associated with higher levels of depression and anxiety, lower general health status, and an increase in healthcare visits (Gierk et al., 2014). In the present study, the scale demonstrated good internal consistency (Cronbach’s α = 0.81; McDonald’s ω = 0.81).

#### 2.2.4. Multidimensional Assessment of Interoceptive Awareness (MAIA)

The Multidimensional Assessment of Interoceptive Awareness (MAIA) is a 32-item self-report measure assessing eight dimensions of interoceptive awareness: noticing, not distracting, not worrying, attention regulation, emotional awareness, self-regulation, body listening, and trusting (Mehling et al., 2012). Responses are rated on a 6-point Likert scale ranging from 0 (never) to 5 (always). Scores for each of the eight MAIA subscales were calculated by averaging items within each subscale. The overall interoceptive awareness score used in the primary analyses was calculated by averaging the MAIA items, with higher scores indicating greater interoceptive awareness. Items in the not distracting and not worrying subscales were reverse-scored prior to calculating subscale scores. In the original validation study, Cronbach’s alpha coefficients ranged from 0.66 to 0.87 across the eight subscales. The MAIA demonstrated evidence of construct validity through factor analyses, known-group comparisons, and associations with established measures of related constructs (Mehling et al., 2012). In the present study, the total MAIA score demonstrated excellent internal consistency (Cronbach’s α = 0.92; McDonald’s ω = 0.93). Among the subscales, noticing demonstrated acceptable internal consistency (α = 0.79; ω = 0.79), whereas not distracting (α = 0.63; ω = 0.64) and not worrying (α = 0.51; ω = 0.61) showed lower internal consistency. Attention regulation (α = 0.91; ω = 0.91), emotional awareness (α = 0.88; ω = 0.87), self-regulation (α = 0.88; ω = 0.87), body listening (α = 0.86; ω = 0.86), and trusting (α = 0.91; ω = 0.92) demonstrated good to excellent internal consistency.

Although the MAIA assesses distinct dimensions of interoceptive awareness, a total score was included to provide an integrated assessment of interoceptive awareness, which was the primary moderator. Given the MAIA’s multidimensional structure, the eight dimensions were also examined separately to account for dimension-specific patterns.

### 2.3. Procedure

Following ethical approval from the Cyprus Aydin University Ethics Committee (2026/07.006), data were collected through participant recruitment platforms. Each participant was compensated upon survey completion. Informed consent was obtained before the study, including the study’s aim, a right to withdraw statement, the estimated survey duration (6–8 min), and the voluntary nature of participation. No identifiable data were collected.

### 2.4. Statistical Analysis

Statistical analyses were conducted using IBM SPSS Statistics version 29 and the PROCESS Macro version 4.2 (Hayes, 2022). Before the main analyses, the normality assumption was evaluated by examining the skewness and kurtosis of all study variables, with visual inspection of their normal Q–Q plots. Multivariate normality was additionally assessed using Mardia’s tests of multivariate skewness and kurtosis for the focal variables in the overall and subscale models. Values within ±2 for skewness and ±7 for kurtosis were considered indicative of acceptable normality (West et al., 1995). Pearson correlation analyses were performed to examine the associations among the study variables.

To examine whether interoceptive awareness moderates the relationship between difficulties in emotion regulation and somatic symptoms, moderation analyses were conducted using PROCESS Macro Model 1. Before the moderation analyses, the predictor and moderator variables were mean-centred and the interaction terms were constructed. In the first analysis, the total MAIA score was examined as the moderator. Subsequently, separate moderation analyses examined the moderating role of each MAIA subscale (i.e., noticing, not distracting, not worrying, attention regulation, emotional awareness, self-regulation, body listening, and trusting). Age, gender, and the presence of chronic physical illness were included as covariates because somatic symptom reporting has been shown to vary according to age (Gierk et al., 2014) and gender (Barsky et al., 2001), while chronic physical illness may contribute directly to the presence and severity of physical symptoms. Chronic physical illness was coded as a binary variable (0 = no; 1 = yes). The significance of the interaction effects was evaluated using a bootstrapping procedure with 5000 resamples to generate bias-corrected 95% confidence intervals (CIs) (Hayes, 2022). Confirmatory factor analyses (CFAs) were conducted in JASP to evaluate the measurement structure of the study measures.

Because eight separate moderation analyses were conducted for the MAIA subscales, a Bonferroni correction was applied to control for Type I errors, resulting in an adjusted significance threshold of *p* < 0.00625 (0.05/8). Accordingly, both the main and interaction effects were considered statistically significant only when their *p*-values were below 0.00625 in the subscale analyses. The moderation analysis using the total MAIA score was treated as the primary analysis. It was therefore evaluated using the conventional significance level of *p* < 0.05. For each significant interaction effect, simple slope analyses were examined to evaluate the association between difficulties in emotion regulation and somatic symptoms at low (16th percentile), moderate (50th percentile), and high (84th percentile) levels of the moderator, as provided by the PROCESS macro.

## 3. Results

### 3.1. Descriptive Statistics

Descriptive statistics for the study variables are presented in Table 1. Difficulties in emotion regulation had a mean score of 40.90 (SD = 14.95); somatic symptoms had a mean score of 9.11 (SD = 6.11); and overall interoceptive awareness had a mean score of 2.84 (SD = 0.77). The MAIA subscale mean values ranged from 2.46 (SD = 1.30) for body listening to 3.31 (SD = 1.33) for trusting. Across all variables, skewness values ranged from −0.53 to 0.80, and kurtosis values ranged from −0.87 to 0.18, indicating that the assumption of normality was met (West et al., 1995). Visual inspection of the Q–Q plots also indicated no substantial deviations from normality. Mardia’s tests on the focal continuous variables, with age excluded as a covariate, indicated significant multivariate skewness in the overall model and all eight subscale models (statistics = 57.542–78.259, all *p* values < 0.001) but nonsignificant kurtosis (statistics = −1.008–0.885, all *p* values > 0.05). Multivariate normality was therefore not supported; the departure was confined to skewness.

### 3.2. Confirmatory Factor Analysis

Confirmatory factor analyses were conducted to evaluate the measurement structure of the MAIA, DERS-16, and SSS-8. The eight-factor MAIA model showed approximately acceptable fit: χ^2^(436) = 1199.72, *p* < 0.001, CFI = 0.90, TLI = 0.89, RMSEA = 0.067, 90% CI [0.062, 0.071], SRMR = 0.07. A hierarchical MAIA model, in which the eight first-order factors loaded onto a single second-order interoceptive awareness factor, was also examined. This model showed mixed fit: χ^2^(456) = 1365.00, *p* < 0.001, CFI = 0.882, TLI = 0.871, RMSEA = 0.071, 90% CI [0.067, 0.075], SRMR = 0.083. Not-Distracting and Not-Worrying loaded negatively on the second-order factor. The five-factor DERS-16 model showed generally acceptable fit: χ^2^(94) = 352.94, *p* < 0.001, CFI = 0.95, TLI = 0.93, RMSEA = 0.084, 90% CI [0.074, 0.093], SRMR = 0.04. The one-factor SSS-8 model also showed generally acceptable fit: χ^2^(20) = 70.23, *p* < 0.001, CFI = 0.94, TLI = 0.91, RMSEA = 0.080, 90% CI [0.060, 0.101], SRMR = 0.04. Overall, the CFAs provided generally acceptable, though not uniformly strong, support for the specified measurement models; however, support for representing the eight MAIA dimensions by a single higher-order factor was comparatively weaker.

### 3.3. Correlation Analysis of Variables

Pearson correlation coefficients among the study variables are presented in Table 2. Difficulties in emotion regulation were positively associated with somatic symptoms (r = 0.53, *p* < 0.001) and negatively associated with overall interoceptive awareness (r = −0.26, *p* < 0.001). Overall interoceptive awareness was not significantly associated with somatic symptoms (r = −0.01, *p* = 0.791). Among the MAIA subscales, noticing (r = 0.24, *p* < 0.001) and emotional awareness (r = 0.16, *p* = 0.001) were positively associated with somatic symptoms, whereas not distracting (r = −0.37, *p* < 0.001), not worrying (r = −0.30, *p* < 0.001), and trusting (r = −0.16, *p* = 0.001) were negatively associated with somatic symptoms. Attention regulation, self-regulation, and body listening were not significantly correlated with somatic symptoms (*p*-values > 0.05). Overall interoceptive awareness was positively associated with all MAIA subscales except not distracting and not worrying.

### 3.4. Regression Diagnostics

Prior to the moderation analyses, multicollinearity was assessed using tolerance and variance inflation factor (VIF) statistics. Across all regression models, tolerance values ranged from 0.810 to 0.996, whereas VIF values ranged from 1.004 to 1.234. All VIF values were well below the recommended cutoff values, and all tolerance values were above acceptable thresholds (Field, 2018), indicating that multicollinearity was not present in the data. Additional regression diagnostics, including Durbin–Watson statistics, standardized residuals, Cook’s distance, linearity, homoscedasticity, and residual normality, are presented in Supplementary Table S1.

### 3.5. Moderation Analysis

A moderation analysis was conducted using PROCESS Model 1 (Hayes, 2022) to examine whether overall interoceptive awareness moderated the association between difficulties in emotion regulation and somatic symptoms while controlling for age, gender, and presence of chronic physical illness. Difficulties in emotion regulation and interoceptive awareness were mean-centred prior to the construction of the interaction term. As shown in Table 3, the overall model was significant, F(6, 384) = 40.67, *p* < 0.001 and explained 38.9% of the variance in somatic symptoms (R^2^ = 0.389).

The interaction between difficulties in emotion regulation and interoceptive awareness was significant (B = 0.069, SE = 0.020, t = 3.51, *p* < 0.001, ΔR^2^ = 0.020, with a change in R^2^ attributable to the interaction), indicating that interoceptive awareness moderated the association between difficulties in emotion regulation and somatic symptoms.

**Figure 1:**
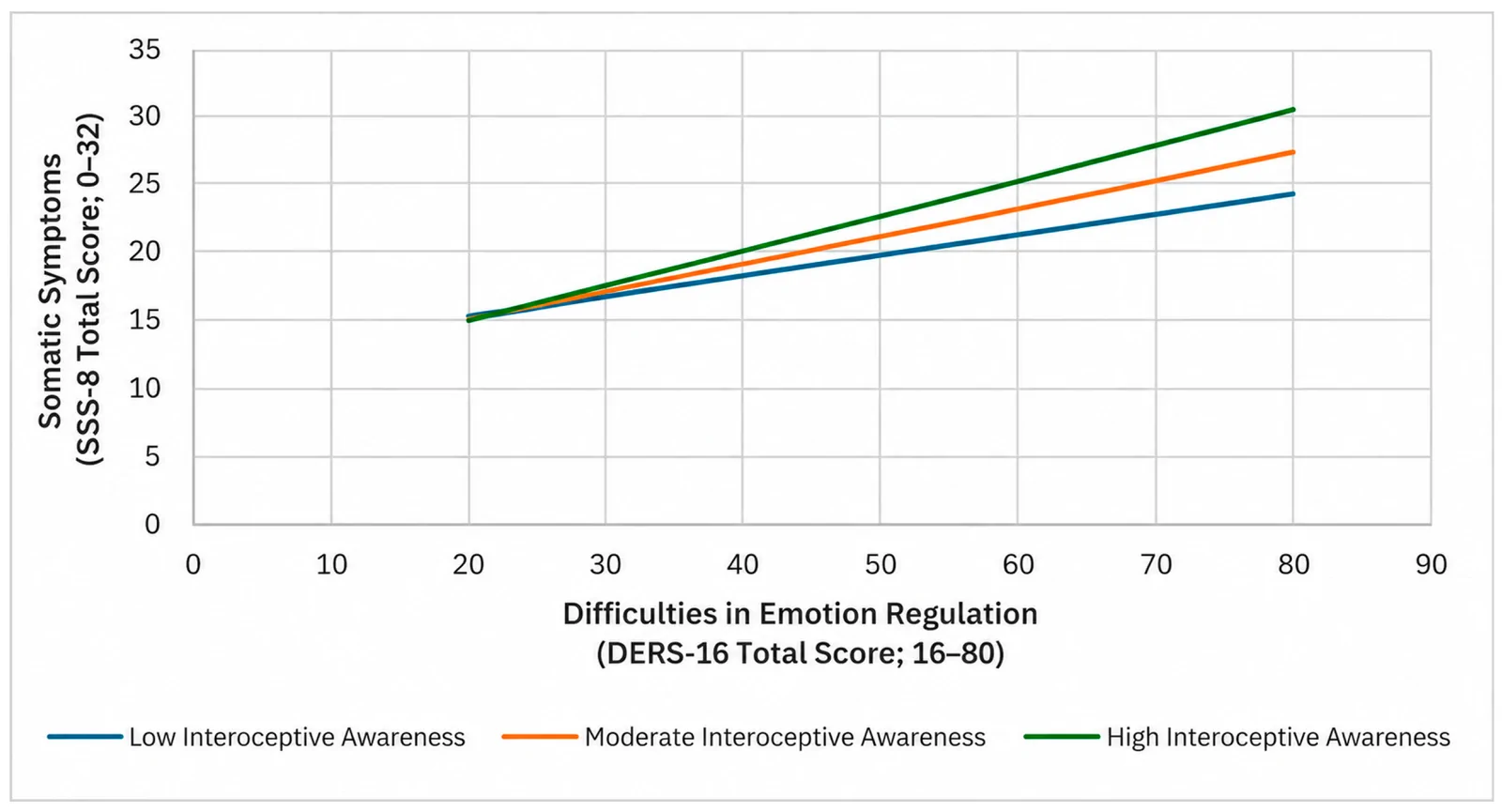
Simple slopes analysis of the interaction between emotion-regulation difficulties and total interoception predicting somatic symptoms.

The simple slope analyses presented in Figure 1 indicate that the positive association between difficulties in emotion regulation and somatic symptoms was significant at low (B = 0.150, SE = 0.023, *p* < 0.001), average (B = 0.204, SE = 0.018, *p* < 0.001), and high (B = 0.256, SE = 0.023, *p* < 0.001) levels of interoceptive awareness. The association became progressively stronger as interoceptive awareness increased, indicating that greater interoceptive awareness amplified the positive relationship between emotion-regulation difficulties and somatic symptoms.

#### Moderation Analysis Examining the Moderator Role of the MAIA Subscales in the Association Between Emotion Regulation Difficulties and Somatic Symptoms

Table 4 presents results for eight separate moderation analyses examining whether the MAIA subscales moderated the association between emotion-regulation difficulties and somatic symptoms, while controlling for gender, age, and chronic physical illness. After applying a Bonferroni correction for multiple comparisons (α = 0.00625), significant interaction effects were observed for noticing (B = 0.039, SE = 0.013, t = 3.02, *p* = 0.003, ΔR^2^ = 0.014), emotional awareness (B = 0.039, SE = 0.014, t = 2.88, *p* = 0.004, ΔR^2^ = 0.013), self-regulation (B = 0.043, SE = 0.012, t = 3.65, *p* < 0.001, ΔR^2^ = 0.021), and body listening (B = 0.038, SE = 0.012, t = 3.23, *p* = 0.001, ΔR^2^ = 0.017). The interaction effects for not distracting (*p* = 0.496), not worrying (*p* = 0.754), attention regulation (*p* = 0.011), and trusting (*p* = 0.087) were not statistically significant after Bonferroni correction.

**Figure 2:**
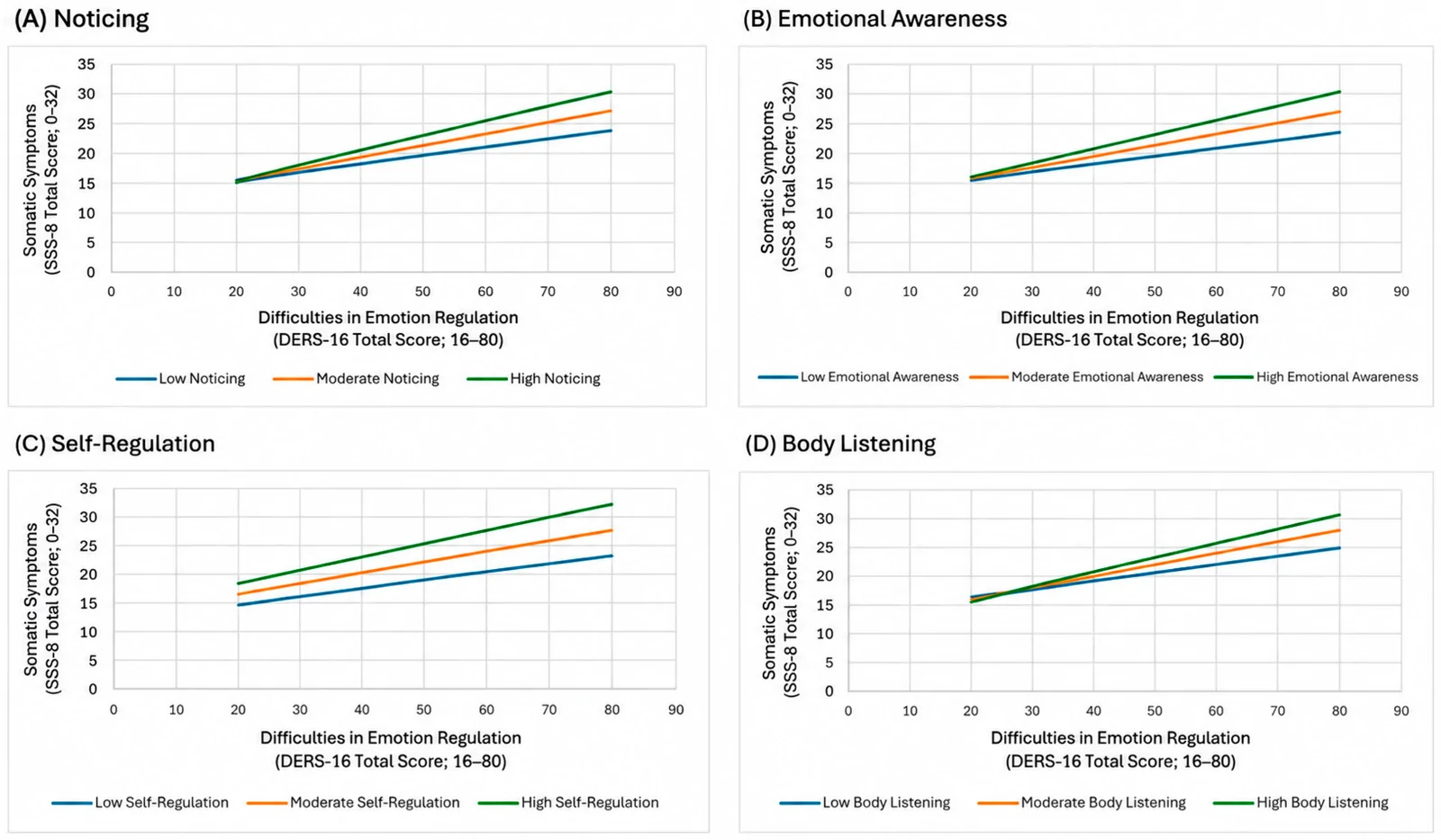
Simple slope analyses of the significant moderating effects of interoceptive awareness dimensions on the association between difficulties in emotion regulation and somatic symptoms: (**A**) noticing; (**B**) emotional awareness; (**C**) self-regulation; and (**D**) body listening. Low, moderate, and high levels of the moderators correspond to the 16th, 50th, and 84th percentiles, respectively.

Simple slope analyses were conducted for the four significant interactions (Figure 2A–D). The positive association between emotion regulation difficulties and somatic symptoms was significant at low, medium, and high levels of noticing, emotional awareness, self-regulation, and body listening (all *p*-values < 0.001). Across all four dimensions, the association became progressively stronger at higher moderator levels, indicating that greater interoceptive awareness within these dimensions strengthened the positive association between emotion regulation difficulties and somatic symptoms.

## 4. Discussion

According to the research findings, greater emotion-regulation difficulties were associated with higher levels of somatic symptoms, a relationship moderated by interoceptive awareness. Among the interoceptive awareness subscales, noticing, emotional awareness, self-regulation, and body listening significantly moderated the association, whereas not distracting, not worrying, attention regulation, and trusting did not. These findings suggest that the link between emotion regulation difficulties and somatic symptoms is stronger among individuals with higher interoceptive awareness.

The correlation analysis revealed a significant positive relationship between emotion regulation difficulties and somatic symptoms. Participants with greater emotion-regulation difficulties also reported more physical symptoms. This finding may be explained by the perseverative cognition hypothesis (Brosschot et al., 2006). According to this framework, prolonged exposure to psychological distress, through maladaptive emotion regulation strategies such as worry and rumination, may extend physiological arousal. Difficulties in emotion regulation may be linked to greater reliance on maladaptive regulatory strategies and may co-occur with prolonged physiological arousal and greater somatic symptom burden. Adaptive emotion regulation requires a series of processes, including monitoring, acceptance, expression, and modulation (Gratz & Roemer, 2004). Lower levels of these skills may be associated with sustained emotional arousal and greater somatic symptom reporting. Previous research has also demonstrated positive associations between emotion regulation difficulties and somatic symptom severity (Mersin et al., 2025; Selvi & Bozo, 2023; Schnabel et al., 2022).

A significant negative relationship was observed between difficulties in emotion regulation and interoceptive awareness. In the literature, interoceptive awareness is associated with effective emotion regulation (Tan et al., 2022; Wadhawan et al., 2025; Füstös et al., 2013). As suggested in the somatic marker hypothesis, bodily sensations may signal changes in the body’s internal state, indicating a need for behavioural or emotional adjustments to maintain homeostasis (Damasio, 1994). Being aware of internal bodily signals associated with emotional experiences may provide insight into the intensity and nature of those experiences. For example, sudden heart palpitations and dizziness may signal increased anxiety. By monitoring and understanding these physical changes, one may be more likely to adopt an adaptive emotion regulation strategy. Furthermore, alleviating these physical symptoms may help regulate underlying emotional experiences. For example, slow-paced breathing practices are shown to ease stress symptoms (Fincham et al., 2023). In contrast, low interoceptive awareness may be linked to emotion regulation difficulties, as understanding and interpreting bodily symptoms are crucial to emotional processing.

A notable finding was that overall interoceptive awareness was not significantly associated with somatic symptom severity at the zero-order level; however, it significantly moderated the association between emotion regulation difficulties and somatic symptoms. Thus, the relevance of interoceptive awareness to somatic symptoms may be conditional rather than direct. Specifically, the association between emotion regulation difficulties and somatic symptoms became stronger at higher levels of interoceptive awareness. One possible explanation is that increased awareness of bodily signals may increase the salience of physiological experiences accompanying emotional dysregulation, making these experiences more noticeable when individuals have difficulty in regulating emotions. In contrast, among individuals with fewer emotion regulation difficulties, greater interoceptive awareness may not necessarily translate into greater symptom reporting. This conditional pattern may help explain inconsistencies in previously reported associations between interoceptive awareness and somatic symptoms, as the role of interoceptive awareness may vary depending on the extent of individuals’ emotion regulation difficulties. Experimental evidence suggests that directing attention toward internal bodily sensations can increase the reporting of bodily sensations (Mirams et al., 2012). This finding may also be considered in light of the distinction between adaptive and maladaptive forms of interoceptive attention. Although interoceptive awareness can facilitate the recognition and regulation of internal states, excessive attention may increase anxiety and the salience of bodily sensations (Trevisan et al., 2021). Although interoceptive awareness is linked to greater emotion regulation (Füstös et al., 2013), it may not necessarily buffer the association between emotion regulation difficulties and somatic symptoms.

The present findings indicate that four specific facets of interoceptive awareness—noticing, emotional awareness, self-regulation, and body listening—moderated the association between emotion regulation difficulties and somatic symptoms, such that higher scores on these subscales strengthened the positive relationship. These findings may be interpreted considering evidence showing that directing attention toward internal bodily sensations can increase the reporting of bodily sensations (Mirams et al., 2012). The noticing subscale reflects awareness of bodily sensations, such as muscle tension and heartbeat. Greater awareness of these sensations during emotion dysregulation may be associated with greater awareness or reporting of somatic symptoms. The emotional awareness subscale highlights the connection between bodily sensations and emotional states. When adaptive emotion regulation skills are lacking, awareness of how bodily sensations relate to emotional states may be associated with greater salience of these sensations. Similarly, body listening, which involves actively listening to the body for insight and guidance, may be related to increased reports of somatic symptoms among individuals with emotion regulation difficulties. Finally, self-regulation, the ability to regulate distress by directing attention toward bodily sensations, also strengthened the association between emotion regulation difficulties and somatic symptoms. Although this dimension is generally considered adaptive, its moderating effect suggests that body-focused regulation may not necessarily weaken the association between emotion regulation difficulties and somatic symptoms.

On the other hand, moderation analysis indicated that the subscales not distracting, not worrying, attention regulation, and trusting did not significantly moderate the association between emotion regulation difficulties and somatic symptoms. Not distracting is the tendency not to ignore or distract oneself from uncomfortable bodily sensations; not worrying is the tendency not to worry when feeling discomfort; attention regulation involves the ability to sustain focus on bodily sensations; and the trusting subscale measures the extent to which one evaluates their body as safe and trustworthy. The pattern of significant and non-significant moderation effects may be interpreted in relation to the five dimensions of the multidimensional conceptual framework for interoceptive awareness proposed by Mehling et al. (2012). Noticing represents awareness of bodily sensations, whereas emotional awareness, self-regulation, and body listening collectively represent awareness of the mind–body integration. All four subscales representing these two dimensions significantly moderated the relationship between emotion regulation difficulties and somatic symptoms. In contrast, the dimensions reflecting emotional and attentional responses to bodily sensations (not distracting and not worrying), the capacity to regulate attention (attention regulation), and trusting bodily sensations (trusting) did not significantly moderate the association. Thus, the findings suggest that the moderating role of interoceptive awareness may be particularly related to the awareness of bodily sensations and their integration with emotional experiences, rather than to how individuals respond to bodily discomfort, regulate attention toward bodily sensations, or experience their body as trustworthy. Greater awareness of bodily sensations and their links to emotional and psychological processes may increase the awareness or reporting of physical sensations in the context of emotion regulation difficulties (Mirams et al., 2012).

Previous research has also indicated that MAIA dimensions may differ in their associations with somatic symptoms. For example, Suzuki et al. (2021) found that noticing, emotional awareness, and body listening were associated with greater somatic symptom severity, whereas not distracting, not worrying, self-regulation, and trusting were associated with lower somatic symptom severity. Similarly, Flasinski et al. (2020) demonstrated that non-distracting and not-worrying were negatively correlated with habitual symptom reporting. Valenzuela-Moguillansky et al. (2017) also found that patients with fibromyalgia reported lower levels of not-distracting and trusting, but higher levels of noticing. In the present study, not-distracting, not-worrying, and trusting were similarly negatively correlated with somatic symptoms, although these dimensions did not significantly moderate the association between emotion regulation difficulties and somatic symptoms. This pattern suggests that some dimensions of interoceptive awareness may be associated with somatic symptom severity without necessarily altering the strength of its relationship with emotion regulation difficulties. Overall, these findings further highlight the importance of considering interoceptive awareness as a multidimensional construct, as its individual dimensions may differ in both their associations with somatic symptoms and their roles in the relationship between emotion regulation difficulties and somatic symptoms.

Several limitations should be considered when interpreting the findings of the present study. First, given the cross-sectional design, a causal interpretation is not possible. Alternative directionalities such as somatic symptoms contributing to emotion-regulation difficulties or bidirectional effects cannot be ruled out. Future longitudinal or experimental designs may clarify temporal precedence and potential causal mechanisms. Secondly, the data are collected using self-report measures, which may be subject to common method bias, social desirability effects and inaccuracies in retrospective reporting. In particular, self-reported interoception does not necessarily correspond to objective interoceptive accuracy. Future research may include behavioural tasks such as heartbeat detection alongside self-report instruments. Third, convenience sampling may have introduced selection bias because participants were not randomly selected, which may limit the representativeness and generalisability of the findings. Future studies should examine whether these findings can be replicated in more diverse populations, including clinical groups such as individuals with somatic symptom disorder and samples from different sociocultural backgrounds. Additionally, the moderation analysis was conducted while controlling for age, gender, and the reported presence of a chronic physical illness. The study did not include other potential confounders, such as personality traits or psychiatric diagnosis. The sample also included a higher proportion of male participants (60.7%), which should be considered when interpreting the findings. Previous research suggests gender differences in interoceptive awareness (Grabauskaitė et al., 2017) and somatic symptoms (Barsky et al., 2001). Therefore, the gender imbalance may have influenced the observed levels of these variables. Although gender was statistically controlled in the analyses, future studies may use a more balanced sample. The low internal consistency of some MAIA dimensions, particularly not worrying (α = 0.51), should also be considered, as low measurement reliability may have attenuated the observed moderation effects. Finally, somatic symptoms were assessed via self-report measures without medical evaluation to distinguish medically explained from medically unexplained symptoms, which may be attributed to emotional distress. Furthermore, the significant interactions accounted for only 1.3–2.1% of additional variance in somatic symptoms, indicating modest effect sizes. Therefore, their practical and clinical significance should be interpreted cautiously. Finally, the comparatively weaker fit of the hierarchical MAIA model also suggests caution when interpreting interoceptive awareness as a unitary construct. Although the overall MAIA score was the primary moderator, the dimension-specific findings better represent the role of interoceptive awareness.

## 5. Conclusions

The study indicates that difficulties in emotion regulation are associated with somatic symptoms, a relationship moderated by interoceptive awareness. Further analysis showed that four dimensions of interoceptive awareness—noticing, emotional awareness, self-regulation, and body listening—strengthened the relationship, whereas not distracting, not worrying, attention regulation, and trusting did not moderate the effect.

Theoretically, the findings suggest that interoceptive awareness may play a more complex role in the relationship between emotion regulation difficulties and somatic symptoms than a protective factor. Specifically, moderating effects were observed for the dimensions of awareness of bodily sensations and awareness of mind–body integration, whereas dimensions reflecting emotional and attentional responses to bodily sensations, regulation of attention, and trust in bodily sensations did not significantly moderate the association. These findings suggest that the theoretical relevance of interoceptive awareness to somatic symptoms may depend on the specific dimensions.

Clinically, the findings may be relevant when considering interventions that encourage increased attention to bodily sensations, such as mindfulness or body-focused practices. In particular, bodily awareness may not be uniformly protective independent of its capacity to regulate emotional distress. Clinicians may therefore consider individual differences in emotion regulation difficulties and interoceptive awareness dimensions when implementing body-focused approaches. However, given the cross-sectional design, non-clinical sample, and relatively small interaction effects, these findings should not be interpreted as evidence that body-focused interventions are associated with increased self-reported somatic symptoms. Longitudinal and intervention studies are needed to determine whether these findings translate into clinically meaningful differences in treatment outcome.

## Figures and Tables

**Table 1:** Descriptive statistics.

	Minimum	Maximum	Mean	Std. Deviation	Skewness	Kurtosis
Difficulties in Emotion Regulation	16	78	40.90	14.95	0.20	−0.75
Somatic Symptoms	0	29	9.11	6.11	0.80	0.18
Interoceptive Awareness	0.72	4.84	2.84	0.77	−0.14	−0.17
Noticing	0	5	2.93	1.16	−0.32	−0.45
Not Distracting	0	5	2.80	1.04	−0.12	−0.41
Not Worrying	0	5	2.55	1.05	−0.13	−0.37
Attention Regulation	0	5	2.76	1.08	−0.16	−0.40
Emotional Awareness	0	5	3.12	1.18	−0.42	−0.38
Self-Regulation	0	5	2.72	1.25	−0.18	−0.71
Body Listening	0	5	2.46	1.30	−0.07	−0.87
Trusting	0	5	3.31	1.33	−0.53	−0.67

**Table 2:** Pearson correlations among the study variables.

Variable	1	2	3	4	5	6	7	8	9	10	11
1. Difficulties in Emotion Regulation	—										
2. Total Interoceptive Awareness	−0.26 ***	—									
3. Somatic Symptoms	0.53 ***	−0.01	—								
4. Noticing	0.06	0.71 ***	0.24 ***	—							
5. Not Distracting	−0.38 ***	−0.01	−0.37 ***	−0.17 ***	—						
6. Not Worrying	−0.37 ***	0.03	−0.30 ***	−0.20 ***	0.11 *	—					
7. Attention Regulation	−0.19 ***	0.87 ***	0.03	0.60 ***	−0.16 **	−0.00	—				
8. Emotional Awareness	0.05	0.80 ***	0.16 **	0.64 ***	−0.18 ***	−0.22 ***	0.63 ***	—			
9. Self-Regulation	−0.24 ***	0.83 ***	−0.02	0.46 ***	−0.13 *	−0.04	0.67 ***	0.63 ***	—		
10. Body Listening	−0.16 **	0.81 ***	0.04	0.53 ***	−0.05	−0.13 **	0.62 ***	0.62 ***	0.70 ***	—	
11. Trusting	−0.34 ***	0.72 ***	−0.16 **	0.37 ***	0.04	0.04	0.52 ***	0.44 ***	0.59 ***	0.63 ***	—

*** *p* < 0.001; ** *p* < 0.01; * *p* < 0.05.

**Table 3:** Moderation analysis examining the moderating role of overall interoceptive awareness in the association between difficulties in emotion regulation and somatic symptoms.

Predictor	*B*	*SE*	*t*	*p*	95% CI
Difficulties in Emotion Regulation	0.204	0.018	11.52	<0.001	[0.169, 0.238]
Interoceptive Awareness	0.932	0.333	2.80	0.005	[0.279, 1.586]
DERS × Interoceptive Awareness	0.069	0.02	3.51	<0.001	[0.030, 0.108]
Gender	−1.197	0.512	−2.34	0.02	[−2.204, −0.190]
Age	−0.066	0.021	−3.08	0.002	[−0.107, −0.024]
Chronic Illness	−3.778	0.636	−5.94	<0.001	[−5.028, −2.528]

Note. B = unstandardised regression coefficient; SE = standard error; CI = confidence interval. The interaction accounted for a significant increment in variance: ΔR^2^ = 0.020, *p* < 0.001.

**Table 4:** Moderating analysis examining the moderating roles of MAIA subscales on the association between emotion-regulation difficulties and somatic symptoms.

MAIA Subscale	B	SE	*t*	*p*	95% CI	ΔR^2^	Bonferroni-Adjusted Significance
Noticing	0.039	0.013	3.02	0.003	[0.014, 0.065]	0.014	Yes
Not Distracting	−0.01	0.015	−0.68	0.496	[−0.040, 0.019]	0.001	No
Not Worrying	−0.005	0.015	−0.31	0.754	[−0.034, 0.025]	<0.001	No
Attention Regulation	0.035	0.014	2.56	0.011	[0.008, 0.062]	0.011	No
Emotional Awareness	0.039	0.014	2.88	0.004	[0.012, 0.066]	0.013	Yes
Self-Regulation	0.043	0.012	3.65	<0.001	[0.020, 0.066]	0.021	Yes
Body Listening	0.038	0.012	3.23	0.001	[0.015, 0.061]	0.017	Yes
Trusting	0.021	0.012	1.72	0.087	[−0.003, 0.044]	0.005	No

Note. B = unstandardised regression coefficient for the interaction term; SE = standard error; ΔR^2^ = change in R^2^ attributable to the interaction. Bonferroni correction was applied for multiple comparisons (adjusted α = 0.00625).

## Data Availability

The data presented in this study are available from the corresponding author upon reasonable request.

## Supplementary Materials

The following supporting information can be downloaded at: https://www.mdpi.com/article/10.3390/bs16101884/s1, Table S1: Regression diagnostics for moderation models examining overall interoceptive awareness and its eight subscales.
