# Supplementary material for: The Moderating Role of Interoceptive Awareness in the Relationship Between Difficulties in Emotion Regulation and Somatic Symptoms
Source: Behav Sci (Basel). 2026 Oct 13;16(10):1884. doi: 10.3390/bs16101884 (PMC13701376; doi:10.3390/bs16101884)
Supplement: Supplementary file 1 [file behavsci-16-01884-s001.zip › behavsci-4563086-supplementary.pdf]

Supplementary Regression Diagnostics

Regression diagnostics were examined for the overall interoceptive awareness model and each subscale moderation model. Across models, tolerance values ranged from .810 to .880 and VIF values ranged from 1.004 to 1.234, indicating no problematic multicollinearity. Durbin–Watson statistics ranged from 1.867 to 1.931. Visual inspection of standardized residual-versus-predicted-value plots indicated no substantial violations of linearity or homoscedasticity, while normal P–P plots indicated no substantial departures from residual normality. Standardized residuals ranged from −3.27 to 3.30 across models. Although several residuals slightly exceeded ±3, Cook's distances were small across all models (maximum = .074), indicating no highly influential observations.

Table S1. Regression diagnostics for moderation models examining overall interoceptive awareness and its eight subscales.

**Moderator**	**Min. Tolerance**	**Max. VIF**	**Durbin–Watson**	**Std. Residual Range**	**Max. Cook's D**	**Linearity / Homoscedasticity**	**Residual Normality**
Overall MAIA	0.849	1.178	1.915	−2.94 to 3.24	0.043	Acceptable	Acceptable
Noticing	0.88	1.136	1.931	−3.27 to 3.08	0.072	Acceptable	Acceptable
Not-Distracting	0.81	1.234	1.877	−2.61 to 2.90	0.072	Acceptable	Acceptable
Not-Worrying	0.815	1.227	1.87	−2.59 to 2.84	0.074	Acceptable	Acceptable
Attention Regulation	0.876	1.141	1.909	−3.20 to 3.11	0.066	Acceptable	Acceptable
Emotional Awareness	0.87	1.15	1.905	−2.52 to 3.23	0.039	Acceptable	Acceptable
Self-Regulation	0.858	1.165	1.895	−2.52 to 3.30	0.042	Acceptable	Acceptable
Body Listening	0.875	1.143	1.867	−2.32 to 3.28	0.056	Acceptable	Acceptable
Trusting	0.824	1.214	1.897	−2.84 to 3.15	0.059	Acceptable	Acceptable
